# Prognostic value of desmoplastic reaction characterisation in stage II colon cancer: prospective validation in a Phase 3 study (SACURA Trial)

**DOI:** 10.1038/s41416-020-01222-8

**Published:** 2021-01-07

**Authors:** Hideki Ueno, Megumi Ishiguro, Eiji Nakatani, Toshiaki Ishikawa, Hiroyuki Uetake, Kenta Murotani, Shigeyuki Matsui, Satoshi Teramukai, Tamotsu Sugai, Yoichi Ajioka, Hirotoshi Maruo, Masahito Kotaka, Masaki Tsujie, Yoshinori Munemoto, Takashi Yamaguchi, Hisashi Kuroda, Mutsumi Fukunaga, Naohiro Tomita, Kenichi Sugihara

**Affiliations:** 1 Department of Surgery, National Defense Medical College, 3-2 Namiki, Tokorozawa, Saitama Japan; 2 Department of Translational Oncology, Tokyo Medical and Dental University, 1-5-45 Yushima, Bunkyo-ku, Tokyo, Japan; 3 Division of Statistical Analysis of Research Support Center, Shizuoka General Hospital, 4-27-1, Kitaando, Aoi-ku, Shizuoka, Japan; 4 Division of Medical Statistics, Translational Research Center for Medical Innovation, Foundation for Biomedical Research and Innovation at Kobe, 1-5-4 Minatojima-minamimachi, Chuo-ku, Kobe, Hyogo, Japan; 5 Department of Specialized Surgeries, Tokyo Medical and Dental University, 1-5-45 Yushima, Bunkyo-ku, Tokyo, Japan; 6 Biostatistics Center, Kurume University, 67 Asahi-Machi, Kurume, Fukuoka, Japan; 7 Department of Biostatistics, Graduate School of Medicine, Nagoya University, 65 Tsurumai-cho, Showa-ku, Nagoya, Aichi Japan; 8 Department of Biostatistics, Kyoto Prefectural University of Medicine, 465 Kajii-cho, Kawaramachi-Hirokoji, Kamigyo-ku, Kyoto, Japan; 9 Department of Molecular Diagnostic Pathology, School of Medicine, Iwate Medical University, 2-1-1, Shiwagun, Yahabachou, Iwate, Japan; 10 Division of Molecular and Diagnostic Pathology, Niigata University Graduate School of Medical and Dental Sciences, 1-757 Asahimachi-dori, Chuo-ku, Niigata, Japan; 11 Department of Surgery, Shizuoka City Shimizu Hospital, 1231 Miyakami, Shimizu-ku, Shizuoka, Japan; 12 Gastrointestinal Cancer Center, Sano Hospital, 2-5-1 Shimizugaoka, Tarumi-ku Kobe, Hyogo, Japan; 13 Department of Surgery, Osaka Prefectural Saiseikai Tondabayashi Hospital, 1-3-36 Koyodai, Tondabayashi, Osaka, Japan; 14 Department of Surgery, Fukui-ken Saiseikai Hospital, 7-1 Funabashi, Wadanaka-cho, Fukui, Japan; 15 Surgery Division, National Hospital Organization Kyoto Medical Center, 1-1 Fukakusa-Mukaihata-cho, Fushimi-ku, Kyoto, Japan; 16 Department of Gastrointestinal Surgery, St. Mary’s Hospital, 422 Tsubuku-honmachi, Kurume, Fukuoka, Japan; 17 Department of Surgery, Sakai City Medical Center, 1-1-1 Ebaraji-cho, Nishi-ku, Sakai City, Osaka, Japan; 18 Division of Lower GI Surgery, Department of Surgery, Hyogo College of Medicine, 1-1 Mukogawa-cho, Nishinomiya, Hyogo, Japan; 19 Tokyo Medical and Dental University, 1-5-45 Yushima, Bunkyo-ku, Tokyo, Japan

**Keywords:** Colon cancer, Cancer microenvironment

## Abstract

**Background:**

The characterisation of desmoplastic reaction (DR) has emerged as a new, independent prognostic determinant in colorectal cancer. Herein, we report the validation of its prognostic value in a randomised controlled study (SACURA trial).

**Methods:**

The study included 991 stage II colon cancer patients. DR was classified by the central review as Mature, Intermediate or Immature based on the presence of hyalinised collagen bundles and myxoid stroma at the desmoplastic front. All clinical and pathological data, including DR characterisations, were prospectively recorded and analysed 5 years after the completion of the registration.

**Results:**

The five-year relapse-free survival (RFS) rate was the highest in the Mature group (*N* = 638), followed by the Intermediate (*N* = 294) and Immature groups (*N* = 59). Multivariate analysis revealed that DR classification was an independent prognostic factor, and based on Harrell’s C-index, the Cox model for predicting RFS was significantly improved by including DR. In the conditional inference tree analysis, DR categorisation was the first split factor for predicting RFS, followed by T-stage, microsatellite instability status and budding.

**Conclusions:**

Histological categorisation of DR provides important prognostic information that could contribute to the efficient selection of stage II colon cancer patients who would benefit from postoperative adjuvant therapy.

## Background

The efficacy of adjuvant treatment in stage II colorectal cancer (CRC) is controversial. The addition of fluorouracil and leucovorin, in comparison with resection alone, exhibited a survival benefit for patients with stage II CRC in both the Quick and Simple and Reliable (QUASAR) trial.^1^ and a pooled analysis of National Surgical Adjuvant Breast and Bowel Project (NSABP) C-01 through C-05.^2^ However, survival analyses of the Surveillance, Epidemiology, and End Results data exhibited no difference in survival with or without adjuvant therapy in stage II colon cancer.^3,4^

In general, patients at high risk of recurrence are believed to derive larger benefits from postoperative chemotherapy than low-risk patients,^5^ and clinical guidelines recommend adjuvant treatment only for ‘high-risk' stage II CRC patients.^6–8^ However, risk definition has not been defined with robust criteria. Currently, stage II CRC patients are considered at high risk of recurrence if they present at least one of the following clinical characteristics: lymph node sampling <12; poorly differentiated tumour, vascular, lymphatic or perineural invasion; tumour presentation with obstruction or tumour perforation; pT4 staging.^6^ All of these are generally regarded as unfavourable for the overall CRC population.

Recent studies have revealed that several genes associated with poor prognosis are expressed by stromal cells rather than by epithelial cancer cells,^9,10^ and it is increasingly recognised that crosstalk between cancer cells and cells of the cancer stroma is involved in the acquired capacity for invasive growth and metastasis.^11,12^ The host response to cancer cells results in the generation of tumour tissue that contains various components, including immune cells, capillaries, activated fibroblasts and extracellular matrix; the growth of connective tissue in this context is known as desmoplastic reaction (DR).^13^ In cancer stroma, also referred to as the tumour microenvironment, cancer-associated fibroblasts (CAFs) are key players that contribute to a wide range of fibrotic remodelling programmes.^11^

The prognostic value of the histological categorisation of DR using haematoxylin and eosin (H&E) staining was first shown in a cohort of rectal cancer patients at St. Mark’s Hospital in the United Kingdom.^14,15^ According to this method, DR of CRC is categorised into three patterns based on specific histological products of CAFs—hyalinised keloid-like collagens and myxoid stroma—found exclusively at the leading edge of the tumour. This method has been applied subsequently to some individual stages of CRC, including T2 CRC,^16^ stage II CRC,^17^ stage III CRC,^18–20^ resectable stage IV CRC^21,22^ and unresectable stage IV CRC,^22^ in which efficient three-tier survival stratification was consistently found using this method. Furthermore, recent multicentre retrospective studies revealed that a significant survival stratification could be achieved by DR categorisation in stage II CRC patients in Japan^23^ and the United Kingdom.^24^ In both of these studies, DR categorisation was shown to be the most significant of all the analysed histopathologic features for predicting survival outcome.

The SACURA trial (Surgical Adjuvant Chemotherapy with UFT for Curatively Resected Stage II Colon Cancer) is a multicentre, randomised controlled study evaluating the superiority of 1-year post-surgical adjuvant treatment with oral tegafur–uracil (UFT) over surgery alone for stage II colon cancer (ClinicalTrials.gov NCT00392899).^25,26^ The 5-year disease-free survival (DFS) rates were 78.4% in the surgery-alone group and 80.2% in the UFT group, respectively (hazard ratio [HR], 0.91; 95% confidence interval (CI), 0.75–1.10; *P* = 0.31). The superiority of adjuvant treatment over surgery alone was not found, although the recurrence rate was lower in the UFT group than in the surgery-alone group (10.4% vs. 13.4%).^26,27^ The SACURA trial projected several translational studies, in which the DR pattern was prospectively evaluated to determine its prognostic value in stage II colon cancer.^25,26^

In this paper, we demonstrated the results of prognostic analyses based on DR categorisation performed 5 years after the completion of patient registration. The main purpose of this study was to prospectively validate the prognostic power of DR categorisation and to elucidate its predictive impact on adjuvant chemotherapy in stage II colon cancer patients.

## Methods

### Patients

In the SACURA trial, after excluding 42 ineligible patients from 2024 patients with stage II colon cancer who were enrolled between October 2006 and July 2010,^25^ 1982 patients were randomly assigned to the surgery-alone group or the surgery plus UFT group (Supplementary Fig. 1). They were assessed for a primary endpoint of DFS and secondary endpoints of overall survival, relapse-free survival (RFS) and incidence and severity of adverse events. At the primary analysis 5 years after the last patient’s enrolment, the trial found no benefit in any of the endpoints for the UFT group.^27^

The translational study for new histopathological prognostic factors was preplanned in the study protocol to include approximately 1000 patients out of those enrolled in the SACURA trial, and consequently, pathological specimens were collected from 1003 eligible patients treated in 123 hospitals (Supplementary Table 1). We excluded 12 patients due to noncompliance with the allocated treatment protocol, and finally, 991 patients with curatively resected stage II colon cancer (surgery-alone group, 501 patients; UFT group, 490 patients) were included in the present study. Of these, 807 patients had colon cancer (caecum, 73; ascending colon, 209; transverse colon, 123; descending colon, 60; sigmoid colon, 342), and 184 had rectosigmoid cancer. The median follow-up was 69.7 (range: 2.1–105.6) months, and the 5-year RFS was 84.2% (surgery-alone group, 83.2%; UFT group, 85.3%).

### Pathological examination for DR characterisation and tumour budding

Postoperatively, glass slides stained with H&E as part of routine pathological practice at each institution were collected in the study office at Tokyo Medical and Dental University. The samples were then submitted to the National Defense Medical College, the institution responsible for central review of new histopathological factors, which was blinded to patient and tumour information. The slides were prepared from whole-tumour sections, which included the deepest part of the tumour, and were prospectively examined by one of the authors (H.U.) in order to evaluate the pattern of DR and tumour budding grade.

**Fig. 1:**
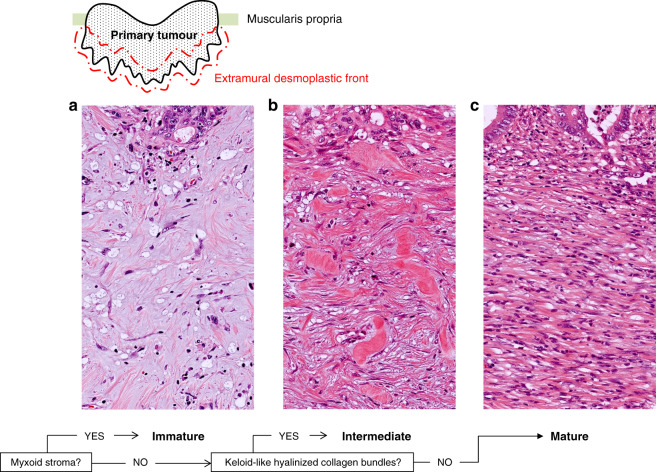
Flowchart for categorising desmoplastic reaction (DR). H&E slides were first scanned at low-power magnification to identify myxoid stroma or keloid-like collagen bundles in the extramural desmoplastic front. The designation of Immature type was applied to tumours in which the largest myxoid stroma completely filled a ×40 objective lens field. If there were no myxoid stroma that met this criterion, DR was categorised as Intermediate or Mature based on whether stroma contained keloid-like collagen or not, respectively. A (myxoid stroma), an amorphous stromal substance comprising basophilic extracellular matrix; B (keloid-like hyalinised collagen bundles), thick bundles of hypocellular collagen with bright eosinophilic hyalinisation; C, Mature-type DR contains neither myxoid stroma nor keloid-like collagen and typically comprises only fine mature collagen fibres stratified into multiple layers.

DR was evaluated according to previous reports^15^ and histologically categorised into three—Immature, Intermediate or Mature—based on the existence of myxoid stroma or keloid-like collagen at the extramural desmoplastic front on H&E-stained slides (Fig. 1). Myxoid stroma is defined as stroma with an amorphous mucinous substance, typically composed of a slightly basophilic or amphophilic, vacuolated extracellular material among the collagen fibres. Keloid-like collagen is characterised by hyalinised thick bundles of hypocellular collagen with bright eosinophilic hyalinisation.

More specifically, H&E slides were first scanned at low-power magnification to identify myxoid stroma and keloid-like collagen in the extramural reactive fibrous area. Tumours with myxoid stroma were designated as Immature. For the minimum amount of myxoid stroma to be judged as Immature, the microscopic field of a ×40 objective lens was used as a yardstick. If there were no myxoid stroma that met the criteria for Immature DR, DR was categorised as Intermediate or Mature, depending on whether the fibrotic stroma contained keloid-like collagen or not, respectively; typically, Mature DR was only composed of fine mature collagen fibres stratified into multiple layers in all reactive fibrous zones. Areas of stroma around microscopic abscesses were not taken into consideration. The Kappa coefficients for intra- and interobserver variations in DR categorisation were previously reported as 0.79^28,29^ and 0.69^28^–0.83,^24^ respectively.

Tumour budding was defined as an isolated cancer cell or cluster comprising <5 cells at the invasive front and graded according to its number in a microscopic field with a ×20 objective lens (0.785 mm^2^) in the hotspot.^26^ We classified tumours with <5, 5–9 and ≥10 budding foci as grades BD1, BD2 and BD3, respectively. These assessment criteria were subsequently adopted in the Japanese Society for Cancer of the Colon and Rectum (JSCCR) guidelines for the treatment of CRC (2009)^7^ and international criteria in the ITBCC 2016.^30^

### Statistical analyses

In the SACURA trial, DFS was defined as the time from randomisation to recurrence, secondary cancers (metachronous cancers developed in both the colorectum and other organs) or death, whichever occurred first.^25,27^ As reported previously,^27^ ~9% of patients experienced secondary cancers, accounting for 40.7% of the DFS events, in the SACURA trial. Thus, we used RFS, the time from randomisation to the first recurrence or death, as a substitute endpoint to evaluate the prognostic value of DR classification, because we considered it to be more suitable for evaluating the clinical value of the prognostic factors.

The Kruskal–Wallis test and the chi-squared test with continuity correction were used for continuous and categorical variables, respectively, to assess the differences in clinicopathological characteristics and recurrence rates according to the patterns of DR. The RFS, as well as time to recurrence (the time from randomisation to the first recurrence), was estimated using the Kaplan–Meier method. We evaluated the 95% CI at a specific time using the standard error computed by Greenwood’s formula and performed comparisons employing the log-rank test. Univariate and multivariate analyses using the Cox proportional-hazards regression model were conducted to calculate HR and 95% CI for RFS of nine prognostic factors. These included six conventional factors used in current international guidelines (number of lymph nodes examined, tumour differentiation, T-stage, lymphatic invasion, venous invasion and microsatellite instability (MSI) status),^6,8^ treatment arm, tumour budding and DR characterisation. We also conducted additional multivariate analyses for other sets of prognostic factors as sensitivity analyses.

Furthermore, we evaluated the difference in Harrell’s C-index between a full Cox model with nine factors and a reduced model with one factor left out from the full model to assess the prognostic power of individual factors.^31^ According to the bootstrap percentile method with resampling (10,000 times), the 95% CI for the difference in Harrell’s C-index from the interest model was calculated. In order to identify prognostic factors with the greatest impacts on risk stratification, we also conducted a conditional inference tree analysis based on a nonparametric binary recursive partitioning^32^ to produce a tree-based stratification with homogeneous subgroups of patients at different risks of RFS.

An interaction analysis was conducted to compare the treatment effects of UFT between subgroups determined by the pattern of DR by using a Cox model, and we also estimated subgroup-specific treatment effects to review the profile of the interactions. All statistical analyses were conducted using the SAS software version 9.4 (SAS Institute Inc., Cary, NC, USA) and R version 3.6.0 (R Foundation, Vienna, Austria).

## Results

### Incidence of each DR category

According to DR characterisation, 638 (64.4%), 294 (29.7%) and 59 (6.0%) patients were classified as Mature, Intermediate and Immature, respectively. The distribution of DR type was significantly associated with tumour differentiation grade, T-stage, tumour budding grade and serum CEA value (*P* ≤ 0.001–0.022), and these were categorised as most unfavourable in the Immature group, followed by the Intermediate and Mature groups (Table 1). In addition, DR type was marginally associated with lymphatic (*P* = 0.0503) and venous invasion (*P* = 0.0580) and tumour location (*P* = 0.0566).

**Table 1:** DR categorisation and clinicopathological characteristics

		DR pattern			
Parameters	Categories	Mature (*N* = 638)	Intermediate (*N* = 294)	Immature (*N* = 59)	*P* value
Gender	Male	386 (60.5)	181 (61.6)	34 (57.6)	0.8460
	Female	252 (39.5)	113 (38.4)	25 (42.4)	
Age (average; years)		65.5	65.5	64.9	0.7373
Tumour location	Rt-sided colon	270 (42.3)	116 (39.5)	19 (32.2)	0.0566
	Lt-sided colon	264 (41.4)	109 (37.1)	29 (49.2)	
	Rectosigmoid	104 (16.3)	69 (23.5)	11 (18.6)	
Maximum diameter (mm)		49.7	47.5	46.1	0.6829
No. of examined LNs (average)		20.5	19.7	18.7	0.4395
No. of examined LNs	≤11	148 (23.2)	77 (26.2)	17 (28.8)	0.4420
	≥12	490 (76.8)	217 (73.8)	42 (71.2)	
Tumour differentiation	G1	292 (45.8)	106 (36.1)	22 (37.3)	0.0211
	G2	321 (50.3)	169 (57.5)	36 (61.0)	
	G3	25 (3.9)	19 (6.5)	1 (1.7)	
T-stage	T3	570 (89.3)	220 (74.8)	33 (55.9)	<0.0001
	T4	68 (10.7)	74 (25.2)	26 (44.1)	
Lymphatic invasion	Negative	286 (44.8)	108 (36.7)	22 (37.3)	0.0503
	Positive	352 (55.2)	186 (63.3)	37 (62.7)	
Venous invasion	Negative	265 (41.5)	98 (33.3)	23 (39.0)	0.0580
	Positive	373 (58.5)	196 (66.7)	36 (61.0)	
Tumour budding	BD1	317 (49.7)	57 (19.4)	2 (3.4)	<0.0001
	BD2	220 (34.5)	102 (34.7)	9 (15.3)	
	BD3	101 (15.8)	135 (45.9)	48 (81.4)	
Preoperative CEA (ng/ml)	≤5.0	459 (71.9)	181 (61.6)	30 (50.8)	<0.0001
	>5.0	152 (23.8)	98 (33.3)	26 (44.1)	
	Not available	27 (4.2)	15 (5.1)	3 (5.1)	
MSI	MSI-high	48 (7.5)	19 (6.5)	2 (3.4)	0.2190
	MSI-low/MSS	573 (89.8)	262 (89.1)	57 (96.6)	
	Not available	17 (2.7)	13 (4.4)	0 (0.0)	
Treatment arm	Surgery-alone	320 (50.2)	144 (49.0)	37 (62.7)	0.1480
	UFT	318 (49.8)	150 (51.0)	22 (37.3)	

*DR* desmoplastic reaction, *LN* lymph node, *CEA* carcinoembryonic antigen, *MSI* microsatellite instability, *MSS* microsatellite stable, *UFT* tegafur–uracil.

### DR category and RFS and recurrence pattern

**Fig. 2:**
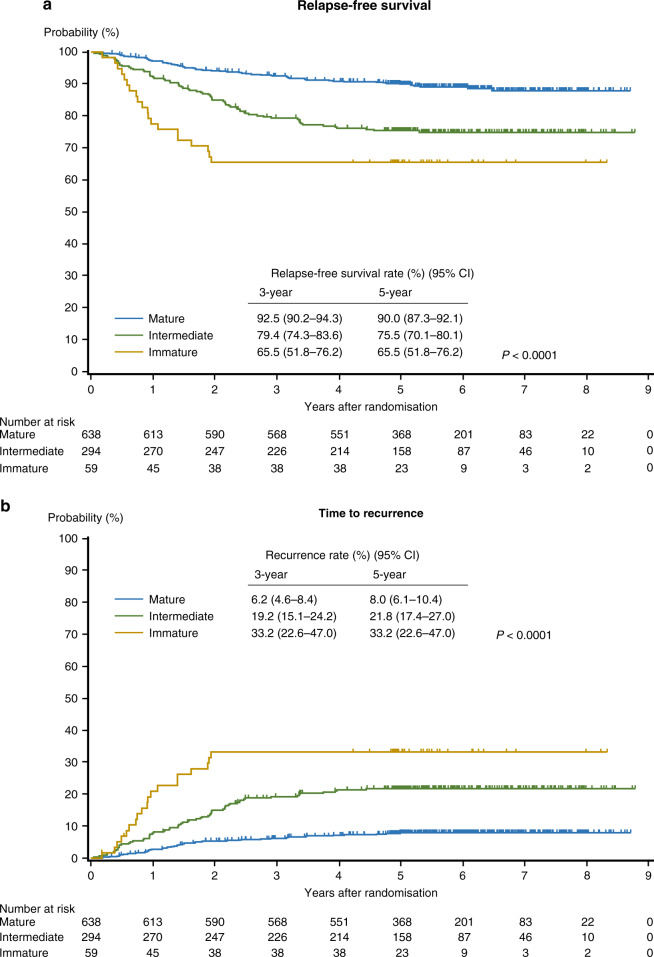
Kaplan–Meier estimates for relapse-free survival (**a**) and time to recurrence (**b**) in stage II colon cancer patients based on the categorisation of desmoplastic reaction.

Five-year RFS rate was the highest in the Mature group (90.0%), followed by the Intermediate (75.5%) and Immature (65.5%) groups (Fig. 2). This trend was similar in the cohort of MSI-stable/low (*N* = 892), in which the 5-year RFS rates were 89.6%, 74.1% and 66.1% for the Mature, Intermediate (HR, 2.6; 95% CI, 1.9–3.7) and Immature groups (HR, 3.9; 2.3–6.6), respectively (*P* < 0.0001). The recurrence rates for these groups 5 years after randomisation were 8.0%, 21.8% and 33.2%, respectively.

A significant impact of DR categorisation on RFS was invariably observed in subset analyses for tumour location (right-sided colon/left-sided colon/rectosigmoid), tumour diameter (≤50 mm/>50 mm), number of lymph nodes examined (≤11/≥12), T-stage (T3/T4), tumour differentiation (G1/G2/G3), lymphatic invasion (negative/positive), venous invasion (negative/positive) and CEA (≤ 5.0 ng/mL/>5.0 ng/mL) (data not shown).

DR type was significantly associated with the number of patients who experienced recurrence in the liver, lung, non-regional lymph node, peritoneum, lymph node and local (*P* ≤ 0.0001–0.027; Supplementary Table 2).

### Univariate and multivariate analyses of prognostic factors for RFS

Univariate analysis using the Cox proportional-hazards regression model showed that, among nine prespecified elemental prognostic factors, T-stage, MSI status, tumour budding and DR characterisation were significantly associated with RFS (Table 2). Multivariate analysis for RFS demonstrated that DR type (Intermediate: HR, 1.85 [95% CI: 1.27–2.69], *P* = 0.0013; Immature: HR, 2.18 (1.23–3.87), *P* = 0.0074) was an independent prognostic factor along with T-stage (T4: HR, 2.19 (1.54–3.13), *P* < 0.0001) and tumour budding (BD2: HR, 1.30 (0.83–2.05), *P* = 0.2529; BD3: HR, 1.87 (1.18–2.97), *P* = 0.0079) (Table 2). In additional multivariate analyses with the other two sets of prognostic factors as sensitivity analyses, DR type was similarly identified as an independent factor for RFS (Supplementary Table 3).

**Table 2:** Univariate and multivariate analyses of RFS using the Cox proportional-hazards regression model.

Parameter	Category	*N*	Univariate		Multivariate^a^	
			HR (95% CI)	*P* value	HR (95% CI)	*P* value
No. of LN examined	≥12	749	1		1	
	<12	242	1.26 (0.90–1.78)	0.1799	1.20 (0.84–1.71)	0.3298
Tumour differentiation	G1	420	1		1	
	G2	526	1.25 (0.91–1.72)	0.1734	1.09 (0.78–1.51)	0.6260
	G3	45	0.45 (0.14–1.44)	0.1808	0.54 (0.16–1.81)	0.3174
T-stage	T3	823	1		1	
	T4	168	2.76 (1.98–3.84)	<0.0001	2.19 (1.54–3.13)	<0.0001
Lymphatic invasion	Negative	416	1		1	
	Positive	575	1.10 (0.80–1.51)	0.5682	0.93 (0.67–1.31)	0.6916
Venous invasion	Negative	386	1		1	
	Positive	605	1.29 (0.93–1.79)	0.1293	1.10 (0.77–1.55)	0.6042
MSI	MSI-low, MSS	892	1		1	
	MSI-high	69	0.33 (0.12–0.90)	0.0296	0.43 (0.15–1.21)	0.1089
Treatment arm	Surgery-alone	501	1		1	
	UFT	490	0.85 (0.62–1.16)	0.3099	0.86 (0.62–1.19)	0.3675
Tumour budding	BD1	376	1		1	
	BD2	331	1.58 (1.03–2.42)	0.0352	1.30 (0.83–2.05)	0.2529
	BD3	284	2.93 (1.97–4.36)	<0.0001	1.87 (1.18–2.97)	0.0079
DR pattern	Mature	621	1		1	
	Intermediate	281	2.51 (1.80–3.51)	<0.0001	1.85 (1.27–2.69)	0.0013
	Immature	59	4.13 (2.50–6.80)	<0.0001	2.18 (1.23–3.87)	0.0074

*RFS* relapse-free survival, *LN* lymph node, *MSI* microsatellite instability, *MSS* microsatellite stable, *UFT* tegafur–uracil, *DR* desmoplastic reaction, *HR* hazard ratio, *CI* confidence interval.

^a^In total, 961 patients with MSI values were analysed.

### Contribution of individual prognostic factors to the improvement of a prognostic model

In Table 3, in terms of Harrell’s C-index, multivariable Cox models for predicting RFS are compared to estimate the contribution of individual prognostic factors. The C-index of a multivariable Cox model consisting of all nine prespecified elemental prognostic factors (full model) was 0.6957. When we compared nine Cox models made by excluding a component factor from the full model, DR type was most associated with a substantially reduced C-index. Specifically, the reduction in C-index was the greatest in the model excluding DR type (0.0196), of which 95% CI was greater than zero (0.0020–0.0449). T-stage was also associated with a significant reduction of the C-index in the prognostic model.

**Table 3:** Comparison of multivariable Cox models for RFS to estimate the contribution of individual prognostic factors according to Harrell’s C-index

Combinations of prognostic factors	Harrell C-index	Difference (reduction) of Harrell C-index (vs. full model)	95% CI of difference
Nine factors (full model)^a^	0.6957	–	–
Eight factors excluding NLNE from the full model	0.6966	−0.0008	−0.0044 to 0.0079
Eight factors excluding tumour differentiation from the full model	0.6941	0.0017	−0.0023 to 0.0143
Eight factors excluding T-stage from the full model	0.6826	**0.0132**	**0.0005 to 0.0329**
Eight factors excluding lymphatic invasion from the full model	0.6940	0.0017	−0.0027 to 0.0084
Eight factors excluding venous invasion from the full model	0.6963	−0.0006	−0.0047 to 0.0081
Eight factors excluding MSI from the full model	0.6881	0.0077	−0.0008 to 0.0198
Eight factors excluding treatment arm from the full model	0.6939	0.0019	−0.0021 to 0.0133
Eight factors excluding tumour budding from the full model	0.6843	0.0114	−0.0015 to 0.0329
Eight factors excluding DR from the full model	0.6761	**0.0196**	**0.0020– 0.0449**

*RFS* relapse-free survival, *NLNE* the number of lymph node examined, *MSI* microsatellite instability, *MSS* microsatellite stable, *UFT* tegafur–uracil, *CI* confidence interval.

Only 961 patients with MSI values were analysed.

Bold values indicates that the factors are associated with a substantially reduced Harrell C-index (95% CI of difference does not contain zero).

^a^Prognostic model consisting of nine elemental prognostic factors {number of lymph nodes examined (<12; ≥12), tumour differentiation (G1; G2; G3), T-stage (T3; T4), lymphatic invasion (negative; positive), venous invasion (negative; positive), MSI (MSI-Low/MSS; MSI-High), treatment arm (surgery alone; UFT), tumour budding (BD1; BD2; BD3), and DR (mature; intermediate; immature)}.

### Tree analysis

**Fig. 3:**
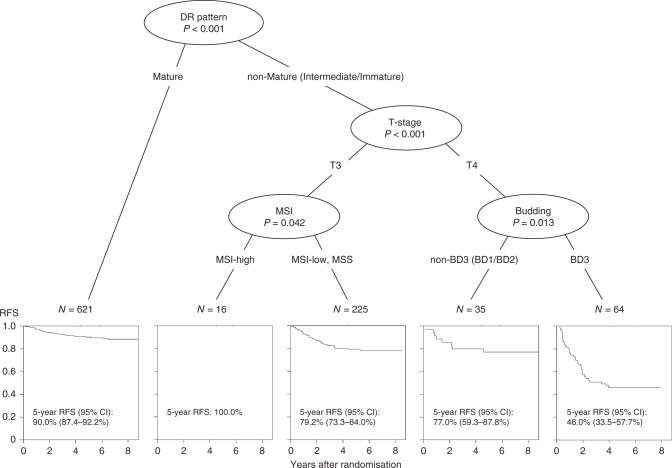
Conditional inference tree analysis to identify the best predictors of RFS and develop a risk stratification model. A total of 961 patients with microsatellite instability values were analysed. DR desmoplastic reaction, MSI microsatellite instability, RFS relapse-free survival, CI confidence interval.

Figure 3 presents the final tree generated by the tree analysis for RFS in 961 patients with microsatellite instability values. Of the nine elemental prognostic factors evaluated, the tree method identified that characterisation of DR as Mature or non-Mature (Intermediate/Immature) was the best single discriminator for RFS, followed by T-stage, MSI status and tumour budding.

### Effect of adjuvant chemotherapy on recurrence prevention according to DR characterisation

The effects of UFT treatment on recurrence prevention were not significantly different between the Mature and non-Mature groups in the interaction test (*P* = 0.944). More specifically, HRs by UFT administration were 0.75 (0.48–1.16) in patients with non-Mature DR and 0.75 (0.43–1.33) in patients with Mature DR. We observed a 5.4% difference in the recurrence rate 5 years after randomisation between the surgery-alone and UFT adjuvant groups, favouring UFT treatment, in patients with non-Mature DR; however, this difference was statistically insignificant (Supplementary Fig. 2).

## Discussion

Recent retrospective multicentre studies revealed that the type of DR in a primary tumour affects postoperative prognostic outcomes independently of tumour stage, and this factor may outweigh conventional tumour factors in terms of the power for predicting RFS in early CRC.^19,23,33^ These results were validated in this study, in which DR characterisation was prospectively determined in stage II colon cancer and its prognostic value was evaluated employing three different statistical approaches. First, based on the Cox proportional-hazard model, DR characterisation was identified as an independent prognostic factor, along with T-stage and tumour budding. The results were similar in the sensitivity analyses with different combinations of prognostic factors. Second, we compared Harrell’s C-index between Cox models with prespecified factors and found that DR characterisation was essential for improving the performance of prognostic predictions.

Third, using conditional inference tree analysis, we attempted to elucidate which factors were essential to produce a decision tree that identified homogeneous subgroups of patients with different likelihoods of RFS. The results revealed that determining whether DR was Mature or non-Mature was the single best discriminator of RFS events, and DR type was the only factor that could define one prognostic subgroup by itself. Taken together, these results indicate that stromal factors are essential for prognostic determination and are consistent with recent molecular biology studies indicating that genes associated with poor prognosis are expressed by stromal cells rather than by epithelial cancer cells.^9,10^

The roles of cancer stroma are complex and multifaceted and cannot be simply defined as helpful or harmful to tumours.^34,35^ The potential for tumour growth and metastasis is determined by how tumour cells orchestrate stromal cells for their benefit through various molecular mechanisms. Keloid-like collagen and myxoid stroma are histological features of fibrotic stroma formed by activated CAFs, but these features may not be cancer-specific and can be induced in other contexts, for example, following inflammatory responses to pathogens. Our results suggest that these two fibrotic components are histological biomarkers that indicate tumour cells have successfully recapitulated the pre-existing stromal remodelling process for their development.

Although the biological mechanisms and pathways leading to morphological desmoplastic diversity remain to be elucidated, we suggest some possible reasons for the prognostic value of DR characterisation, which is reported not only in CRC patients but in patients with other types of malignancy, including pancreatic ductal adenocarcinoma,^36^ cutaneous squamous cell carcinoma,^37^ cervical squamous cell carcinoma^38^ and intrahepatic cholangiocarcinoma.^39^ First, it is possible that DR is associated with the epithelial-to-mesenchymal transition (EMT) of neoplastic cells, an idea supported by previous studies.^22^ For example, keloid-like collagen and myxoid stroma are rarely observed at the centre of tumours but rather appear exclusively at the invasive front of tumours, where remodelling of the cancer environment occurs and tumour EMT-like histology is often observed in highly malignant tumours. One extensively documented property of CAFs is the ability to induce EMT in neoplastic cells and thus favours invasiveness.^11^ Our above-mentioned hypothesis is in agreement with the results of our present and previous studies,^15,29,33^ which indicate a close link between DR and the degree of tumour budding, a process closely associated with EMT-related gene expression.^40,41^

The thick and hyalinised collagen bundles that characterise Intermediate DR are also observed in keloids, in which fibroblasts overexpress various growth factors, including transforming growth factor β (TGF-β).^42^ In the tumour microenvironment surrounding these bundles, TGF-β, a well-established inducer of EMT,^43,44^ is highly likely to be upregulated. In addition, the myxoid stroma that characterises Immature DR is associated with excessive deposition of extracellular matrix components, such as fibronectin,^29^ which are known to affect various pro-tumour functions, including EMT activation.^45^ Consequently, a likely mechanism for the adverse prognostic impact of non-Mature DR is that it reflects activated EMT, which promotes carcinoma progression through a variety of mechanisms, including endowing cells with migratory and invasive properties.^46^

Histological features of the stroma associated with unfavourable DR type include fewer immune cells (including tumour-infiltrating T cells and tumour-associated macrophages),^15,17,28,29^ inhibited Crohn’s-like lymphoid reaction^29^ and decreased microvascularity.^29^ Thus, a second possible mechanism for the aggressiveness of CRC with unfavourable DR type is that the cancer stroma and CAFs actively participate in the modulation of immune responses to help neoplastic cells escape detection.^13,47^ Some specific components that provide a structural framework for cancer stroma with Immature DR, e.g., fibronectins and tenascin-C,^29^ may physically interfere with immune cell infiltration, thereby promoting tumour progression.

Identifying high-risk patients by molecular profiling is receiving increased attention, and it has been reported that genetic risk stratification is valuable in the treatment of stage II colon cancer. Using the Oncotype DX colon cancer multigene test, the QUASAR study partitioned risk of recurrence at 3 years into low-, intermediate- and high-risk groups, at 12%, 18% and 22%, respectively.^48^ Similarly, estimates of 5-year recurrence risk were 12%, 15% and 18% in the GALGB 9581 study^5^ and 9%, 14% and 19% in the SUNRISE study.^49^

On the other hand, our previous retrospective multicentre study found that DR categorisation stratified risk of recurrence for two independent cohorts over a wider range in stage II CRC patients; specifically, the cumulative recurrence rate 5 years after surgery ranged between 9–31% in the first cohort and 11–37% in the second cohort.^23^ Given that these figures are similar to those presented here, where there is also a clear differentiation in the recurrence rates among the three prognostic groups (8%, 21% and 32% for Mature, Intermediate and Immature, respectively), it is worth revisiting the classic and H&E-oriented surgical pathology when new treatment-deciding factors based on the role of cancer microenvironment emerge. These may include tumour budding^26^ and DR pattern, which could improve upon current genetic staging in terms of cost–benefit performance.

There is little agreement regarding the benefits of adjuvant therapy in stage II colon cancer. However, given that stage III patients experience greater survival with adjuvant therapy, the benefits of adjuvant therapy could be proportional to the degree of increased risk of recurrence.^50,51^ This scenario may be consistent with the finding of the SACURA trial, in which the values of adjuvant chemotherapy were compared between high- and low-risk groups based on new histological prognostic factors. Specifically, as we previously reported,^26^ there was an improvement in the 5-year recurrence rate in the UFT group of approximately 5% compared with the surgery-alone group in patients with a higher grade of budding. Similarly, we observed in the present study that the proportional reduction in recurrence with chemotherapy in patients with non-Mature DR appears equivalent to that in patients with Mature DR, indicating that absolute reductions in recurrence with adjuvant chemotherapy were more than twice as large in patients with non-Mature DR than in patients with Mature DR. However, these results were not statistically significant, possibly due to the limited number of patients included and the insufficient efficacy of UFT monotherapy.

The effectiveness of orally administered UFT monotherapy has been demonstrated in stage III rectal cancer patients,^52^ and before 5-fluorouracil (5-FU) and leucovorin became standard in clinical practice, UFT monotherapy was widely used as an adjuvant chemotherapy for early CRC in Japan. Currently, the effectiveness of UFT monotherapy is generally considered to be lower than that of current standard regimens, such as 5-FU and leucovorin, UFT and leucovorin, capecitabine and oxaliplatin-based regimens.^7^ A prospective Phase 3 trial conducted by the Japanese Clinical Oncology Group [JCOG1805 (jRCTs031190186): Randomised controlled study of adjuvant chemotherapy for stage II CRC patients at high risk of developing recurrence according to T-stage and three selected pathological factors (Pn, DR and BD); acronym, PanDRa-BD] was initiated in January 2020, in which the predictive value of both DR characterisation and tumour budding in adjuvant chemotherapy settings using these modern regimens will be elucidated.

In conclusion, this study validated the prognostic importance of DR categorisation for stage II colon cancer, an idea that is supported by recent findings emphasising the role of CAFs in the cancer microenvironment. Further attempts to better understand desmoplastic heterogeneity will help in elucidating the link between cancer biology and surgical pathology, resulting in a heightened value of adjuvant chemotherapy in stage II colon cancer.

## Supplementary information

Supplemental material

## Data Availability

All data generated or analysed during this study are included either in this paper or in the supplementary information.
