# Supplementary material for: Prognostic value of desmoplastic reaction characterisation in stage II colon cancer: prospective validation in a Phase 3 study (SACURA Trial)
Source: Br J Cancer. 2021 Jan 7;124(6):1088–97. doi: 10.1038/s41416-020-01222-8 (PMC7960987; doi:10.1038/s41416-020-01222-8)
Supplement: Supplementary file 1 — Supplemental material [file 41416_2020_1222_MOESM1_ESM.pdf]

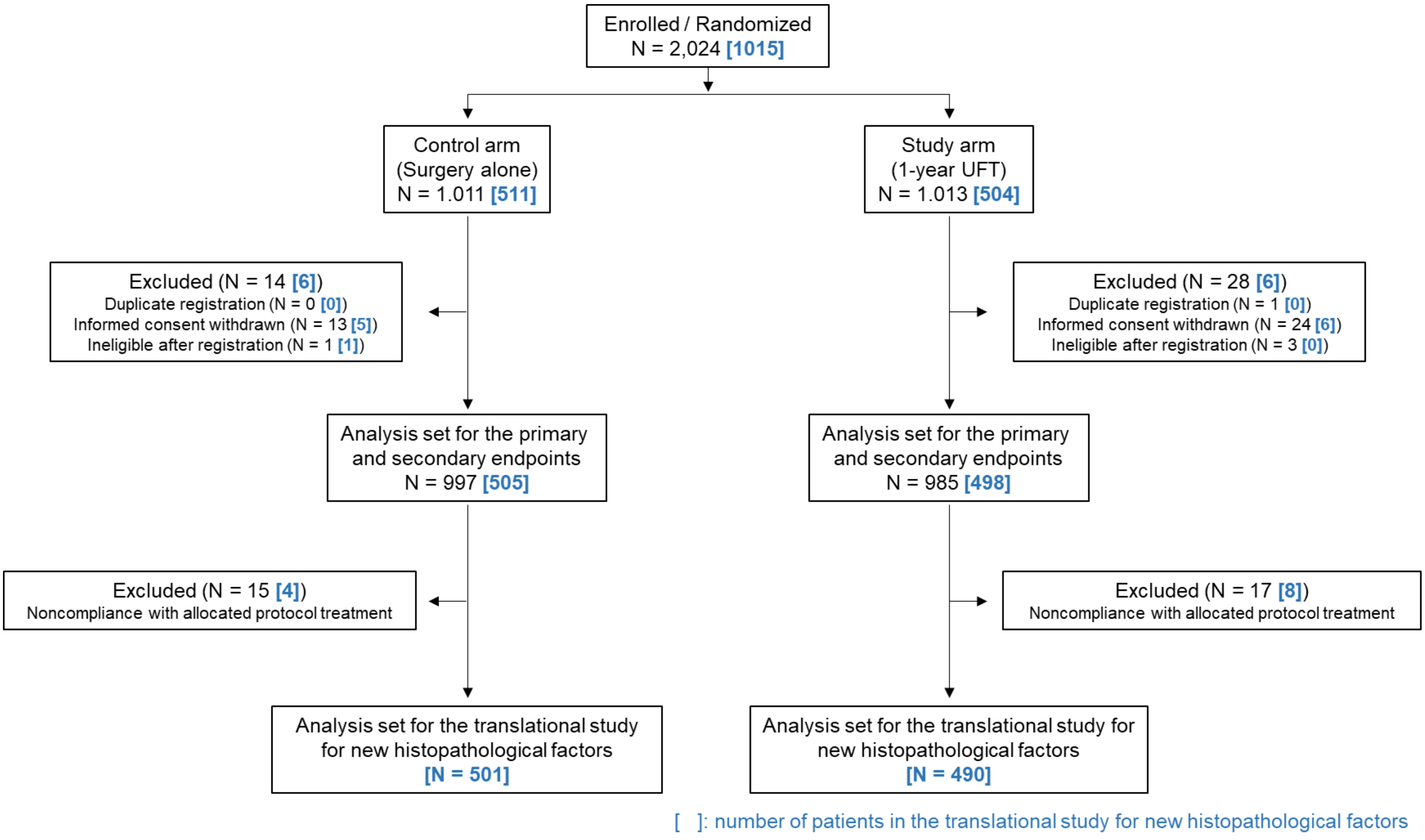

Supplemental Figure 1. CONSORT diagram

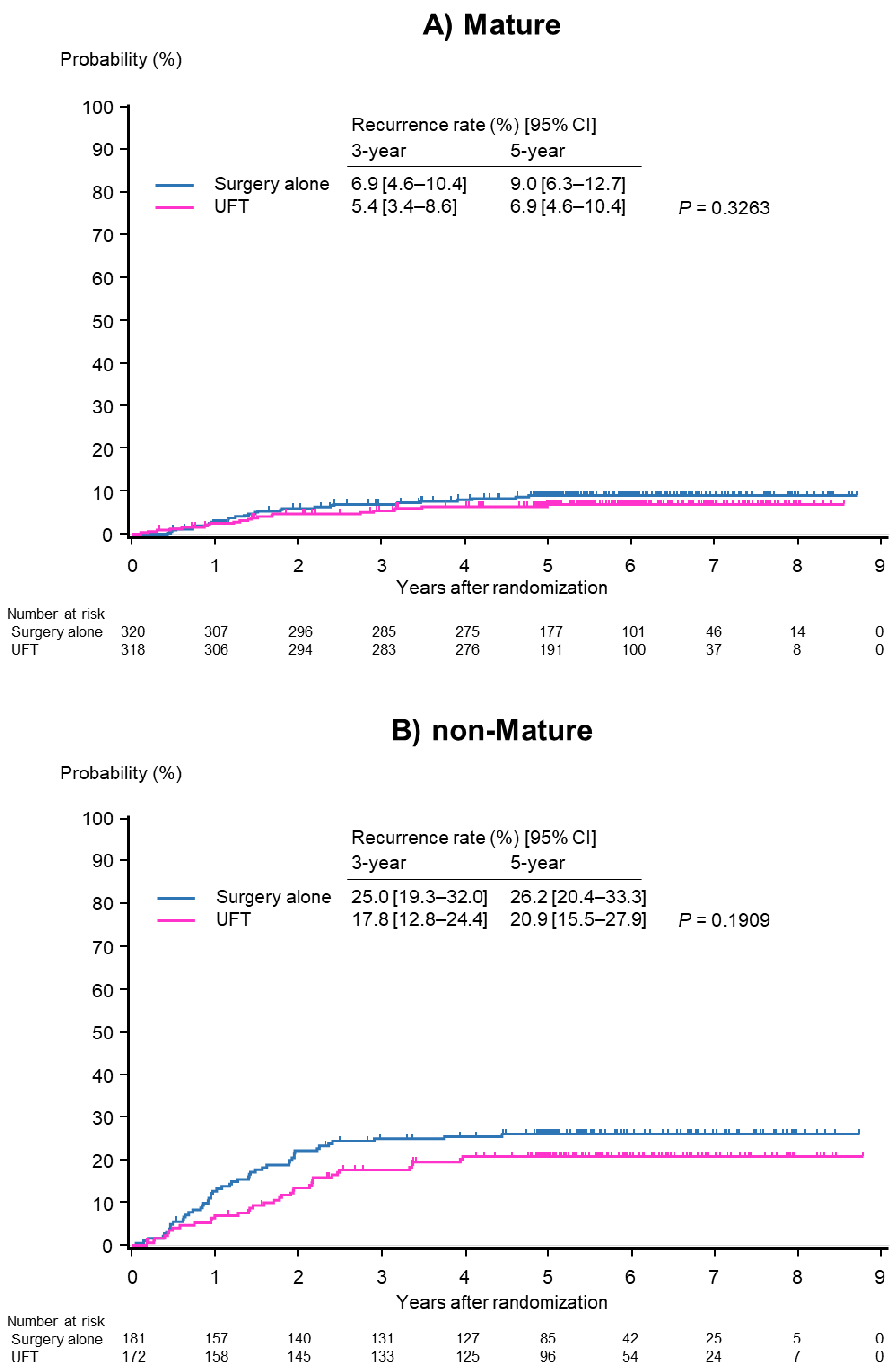

Supplemental Figure 2. Comparison of time to recurrence between the surgery-alone group and the chemotherapy group in A) patients with tumors classified as Mature pattern and B) those with tumors classified as non-Mature pattern.

The 5-year recurrence rates (95% CI) for surgery-alone and the UFT groups were 9.0% (6.3%–12.7%) and 6.9% (4.6%–10.4%) in the Mature group and 26.2% (20.4%–33.3%) and 20.9% (15.5%–27.9%) in the non-Mature group, respectively. UFT, tegafur–uracil

Supplemental Table 1.

A list of institutions that participated in the translational study for new histopathological prognostic factors in the SACURA trial

1. Osaka General Medical Center
2. National Defense Medical College
3. Kobe City Hospital Organization Kobe City Medical Center West Hospital
4. Koseiren Takaoka Hospital
5. Hamamatsu University School of Medicine
6. Saiseikai Yokohamashi Tobu Hospital
7. Tokyo Medical and Dental University
8. JCHO Osaka Hospital
9. Suita Municipal Hospital
10. Japanese Red Cross Kyoto Daini Hospital
11. Nippon Medical School Chiba Hokusoh Hospital
12. Shizuoka City Shimizu Hospital
13. Sano Hospital
14. Saiseikai Tondabayashi Hospital
15. Fukui-ken Saiseikai Hospital
16. National Hospital Organization Kyoto Medical Center
17. St. Mary's Hospital
18. Sakai City Medical Center
19. Ogaki Municipal Hospital
20. Tokyo Metropolitan Tama Medical Center
21. Social Insurance Tagawa Hospital
22. Chugoku Central Hospital
23. Teikyo University School of Medicine
24. National Hospital Organization Kobe Medical Center
25. Hakodate Goryoukaku Hospital
26. Gunma Prefectural Cancer Center
27. Hyogo College of Medicine
28. Kagawa Prefectural Central Hospital
29. International Goodwill Hospital
30. Kobe University Graduate School of Medicine
31. Rinku General Medical Center
32. Kyorin University
33. Niigata Cancer Center Hospital
34. Osaka Police Hospital
35. Kansai Rosai Hospital
36. National Center for Global Health and Medicine
37. Fukushima Medical University
38. Osaka Rosai Hospital
39. Sapporo Medical University
40. Miyoshi Central Hospital
41. Nagoya University Hospital
42. Osaka City General Hospital
43. Higashi Takarazuka Satoh Hospital
44. Tokyo Yamate Medical Center
45. Takarazuka City Hospital
46. Kurashiki Central Hospital
47. kurume university school of medicine
48. Hakodate Municipal Hospital
49. Tochigi Cancer Center
50. Kitakyushu Municipal Medical Center
51. Hashima City Hospital
52. Kure Medical Center and Chugoku Cancer Center
53. Hokushin General Hospital
54. Miyagi Cancer Center
55. Yamagata University Hospital
56. Yamaguchi University Graduate School of Medicine
57. Oita University Graduate School of Medicine
58. Nagano Municipal Hospital
59. Shimonoseki Medical Center
60. Himeji St. Mary's Hospital
61. Nagoya Ekisaikai Hospital
62. Tokushima University Hospital
63. Anan Kyoei Hospital
64. Kumamoto University
65. National Hospital Organization Shikoku Cancer Center
66. Kanagawa Cancer Center
67. Matsunami General Hospital
68. Otemae Hospital
69. Japanese Red Cross Osaka Hospital
70. Hyogo Cancer Center
71. Aichi Cancer Center Aichi Hospital
72. University of Yamanashi Hospital
73. Jichi Medical University Hospital
74. Otsu City Hospital
75. Oita Red Cross Hospital
76. Teikyo University Chiba Medical Center
77. Tohoku Rosai Hospital
78. Kitasato University East Hospital
79. Minoh City Hospital
80. Asahikawa Medical University
81. Kyushu University Graduate School of Medical Sciences
82. Nissay Hospital
83. Gunma University Graduate School of Medicine
84. Nagahama City Hospital
85. Shinshu University Hospital
86. Japanese Red Cross Society Nagano Hospital
87. Tottori University Hospital
88. Kyoritsu General hospital
89. Saitama Medical University International Medical Center
90. Numazu City Hospital
91. Nishijin Hospital
92. Shizuoka City Shizuoka Hospital
93. Fujita Health University Banbuntane Hotokukai Hospital
94. Hokkaido chuo rosai hospital
95. Heisei Yokohama Hospital
96. Iida Municipal Hospital
97. Gifu City Hospital
98. Hiraka General Hospital
99. Tokyo Women's Medical University Hospital
100. University of Fukui Hospital
101. Midori Municipal Hospital
102. Kawakita General Hospital
103. Chigasaki Municipal Hospital
104. Minamiosaka Hospital
105. Saiseikai Hiroshima Hospital
106. Kobe City Nishi-Kobe Medical Center
107. Nanpuh Hospital
108. Noshiro Kousei Medical Center
109. Niigata University Medical & Dental Hospital
110. Hoshigaoka Medical Center
111. Almeida Memorial Hospital
112. Kagawa Rosai Hospital
113. Japanese Red Cross Wakayama Medical Center
114. Kansai Medical University Medical Center
115. Saitama Medical Center
116. Himeji Central Hospital
117. Katsushika Edogawa Hospital
118. Matsuda Hospital
119. International University of Health and Welfare, Mita Hospital
120. National Hospital Organization Kumamoto Minami Hospital
121. Saitama Medical Center
122. Ishikawa Prefectural Central Hospital
123. Saiseikai Nara Hospital

Supplemental Table 2.

The incidence of postoperative oncological events according to the DR pattern

Oncological event	Organ of recurrence	DR pattern Mature (N=638)	Intermed. (N=294)	Immature (N=59)	*P* value
**Recurrence**	Overall	49 (7.7)	63 (21.4)	19 (32.2)	<0.0001
	Liver	*27 (4.2)*	*29 (9.9)*	*4 (6.8)*	*0.0035*
	Lung	*13 (2.0)*	*14 (4.8)*	*10 (16.9)*	*<0.0001*
	Non-regional lymph node	*4 (0.6)*	*8 (2.7)*	*5 (8.5)*	*0.0002*
	Peritoneum	*4 (0.6)*	*10 (3.4)*	*5 (8.5)*	*<0.0001*
	Local	*3 (0.5)*	*7 (2.4)*	*1 (1.7)*	*0.0271*
**Second cancer**		64 (10.0)	21 (7.1)	2 (3.4)	0.1310

DR, desmoplastic reaction

Supplemental Table 3. Multivariate analyses for RFS as sensitivity analysis

Parameter	Category	Selected prognostic factors (N = 991)		Full prognostic factors** (N = 916)	
		HR (95% CI)	*P* value	HR (95% CI)	*P* value
Sex	Female			1	
	Male			1.21 [0.85–1.72]	0.2912
Age (average; years)	≤70			1	
	71–80			1.41 [1.00–2.00]	0.0488
Tumor location	Right-sided colon			1	
	Left-sided colon			1.02 [0.70–1.48]	0.9300
	Rectosigmoid			1.07 [0.67–1.71]	0.7694
Extent of LN dissection*	D3			1	
	D1, D2			1.51 [1.02–2.24]	0.0380
No. of LN examined	≥12	1		1	
	<12	1.27 [0.90–1.80]	0.1715	1.11 [0.76–1.63]	0.5995
Tumor differentiation	G1	1		1	
	G2	1.15 [0.83–1.60]	0.3914	1.08 [0.76–1.52]	0.6762
	G3	0.39 [0.12–1.26]	0.1162	0.70 [0.21–2.38]	0.5650
T-stage	T3	1		1	
	T4	2.14 [1.51–3.04]	<.0001	2.19 [1.52–3.16]	<.0001
Lymphatic invasion	Negative	1		1	
	Positive	0.92 [0.66–1.27]	0.5980	0.97 [0.68–1.37]	0.8391
Venous invasion	Negative	1		1	
	Positive	1.15 [0.82–1.62]	0.4210	1.04 [0.72–1.49]	0.8499
Preoperative CEA (ng/ml)	≤5.0			1	
	>5.0			1.38 [0.98–1.96]	0.0672
MSI	MSI-Low, MSS			1	
	MSI-High			0.34 [0.10–1.14]	0.0801
Treatment arm	Surgery-alone	1		1	
	UFT	0.84 [0.62–1.16]	0.2872	0.91 [0.65–1.27]	0.5821
Tumor budding	BD1	1		1	
	BD2	1.27 [0.82–1.97]	0.2884	1.34 [0.84–2.13]	0.2195
	BD3	1.81 [1.15–2.85]	0.0102	1.98 [1.23–3.19]	0.0049
DR pattern	Mature	1		1	
	Intermediate	1.85 [1.28–2.67]	0.0011	1.76 [1.19–2.59]	0.0046
	Immature	2.21 [1.25–3.89]	0.0063	2.15 [1.20–3.84]	0.0096

RFS, relapse-free survival; LN, lymph node; CEA, carcinoembryonic antigen; MSI, microsatellite instability; MSS, microsatellite stable; UFT, tegafur–uracil; DR, desmoplastic reaction; HR, hazard ratio; CI, confidence interval; *Japanese Classification of Colorectal Carcinoma (Second English Edition); **Patients with CEA and MSI values were analyzed.
